# Identification of Risk Factors for Relapse in Childhood Leukemia Using Penalized Semi-parametric Mixture Cure Competing Risks Model

**DOI:** 10.34172/jrhs.2024.150

**Published:** 2024-06-01

**Authors:** Zahra Mehrbakhsh, Leili Tapak, Nasser Behnampour, Ghodratollah Roshanaei

**Affiliations:** ^1^Department of Biostatistics, School of Public Health, Hamadan University of Medical Sciences, Hamadan, Iran; ^2^Student Research Committee, Hamadan University of Medical Sciences, Hamadan, Iran; ^3^Modeling of Noncommunicable Diseases Research Center, Hamadan University of Medical Sciences, Hamadan, Iran; ^4^Department of Biostatistics and Epidemiology, School of Health, Golestan University of Medical Sciences, Gorgan, Iran

**Keywords:** Mixture cure models, Competing risks, Penalized method, Smoothly clipped absolute deviation, LASSO, Leukemia, Relapse

## Abstract

**Background:** Leukemia is the most common childhood malignancy. Identifying prognostic factors of patient survival and relapse using more reliable statistical models instead of traditional variable selection methods such as stepwise regression is of great importance. The present study aimed to apply a penalized semi-parametric mixture cure model to identify the prognostic factors affecting short-term and long-term survival of childhood leukemia in the presence of competing risks. The outcome of interest in this study was time to relapse.

**Study Design:** A retrospective cohort study.

**Methods:** A total of 178 patients (0‒15 years old) with leukemia participated in this study (September 1997 to September 2016, followed up to June 2021) at Golestan University of Medical Sciences, Iran. Demographic, clinical, and laboratory data were collected, and then a penalized semi-parametric mixture cure competing risk model with smoothly clipped absolute deviation (SCAD) and least absolute shrinkage and selection operator (LASSO) regularizations was used to analyze the data.

**Results:** Important prognostic factors of relapse patients selected by the SCAD regularization method were platelets (150000‒400000 vs.>400000; odds ratio=0.31) in the cure part and type of leukemia (ALL vs. AML, hazard ratio (HR)=0.08), mediastinal tumor (yes vs. no, HR=16.28), splenomegaly (yes vs. no; HR=2.94), in the latency part. In addition, significant prognostic factors of death identified by the SCAD regularization method included white blood cells (<4000 vs.>11000, HR=0.25) and rheumatoid arthritis signs (yes vs. no, HR=5.75) in the latency part.

**Conclusion:** Several laboratory factors and clinical side effects were associated with relapse and death, which can be beneficial in treating the disease and predicting relapse and death time.

## Background

 Leukemia, caused by abnormal hematopoietic cells, is the most common childhood malignancy, with a high mortality rate attributed to ineffective treatment.^1^ There are two main types of childhood leukemia. Acute lymphoblastic leukemia (ALL) and acute myeloid leukemia (AML) contain 80% and 15% of the cases, respectively.^2^ Approximately 31% of all leukemias happen in children under the age of 15.^1^ According to GLOBOCAN 2020, leukemia accounts for 2.5% of new cases and 3.1% of cancer-related deaths among various types of cancer in 185 countries for all age groups.^3^ In leukemia, patients may experience complete remission, relapse, and death.^4^ Despite various treatment options, such as radiotherapy and chemotherapy, relapse of the disease remains common and can lead to death.^5,6^ High rates of complete remission (90%) and 5-year relapse-free survival (75%) have been reported in children with ALL,^1,2^ and AML has shown a 90% complete remission rate.^5^

 Identifying prognostic factors for leukemia survival is crucial due to the high costs and impact on families and healthcare systems. Advanced statistical methods can help predict survival and relapse times for patients.^5,6^ The Cox-PH model is widely used for single-event analysis in survival data. Cause-specific or sub-distribution hazard models are recommended in cases of multiple events or competing risks.^7,8^ In addition, advancements in disease treatment or early diagnosis can lead to longer survival times or remission, making standard survival models such as Cox-PH inappropriate for analysis in such cases.^9,10^ As a proportion of patients do not experience the event of interest, the censoring rate increases, and a Cox-PH model tends to underestimate the hazard and overestimate the survival for the susceptible subjects.^11^

 Researchers in survival studies aim to identify a subset of predictor variables that accurately predict the response variable with less error.^12^ These studies have utilized classical variable selection methods, including best subset selection (BSS)^13^ and stepwise regression^14^ which are easy to use but have some limitations. The BSS method is not practical for a large number of predictor variables due to computational challenges and instability.^15,16^ Stepwise regression faces the problem of “over-fitting”.^17^ The computational intensity of the mixture cure models makes this method impractical due to the inclusion of two regression components within the model. In addition to the computational disadvantage, the stability of the BSS is compromised.^18^

 Recently, penalized regression methods have received much interest from researchers. In penalized regression, a penalty function on the coefﬁcients is added to the likelihood function; accordingly, variable selection and estimation are performed simultaneously.^19,20^ According to the literature review, regularized techniques have been used in the Cox-PH regression,^21^ competing risks regression,^22,23^ and cure models.^18,24,25^ Despite the development of penalty-based methods for variable selection in various survival data, penalty-based methods for variable selection have not been applied in cure competing risk models. Hence, in the present study, a penalized mixture cure competing risk model with a cause-specific approach was utilized to identify the prognostic factors affecting short- and long-term survival in patients with leukemia.

## Methods

 A dataset containing information on 178 patients with ALL and AML who were younger than 15 years and had comprehensive medical records was employed in this retrospective cohort study. The participants were referred to Taleghani Hospital in Gorgan, Iran, from September 1997 to September 2016 and followed up until June 2021. The data related to required factors were available in the dataset; they included demographic information such as age at diagnosis (year), gender (female/male), date of diagnosis, date of death, and date of relapse, as well as laboratory information such as type of leukemia (ALL/AML), white blood cell count at diagnosis (WBC, cells/mL), hemoglobin (Hb, g/dL), and platelets (PLT, cells/mL). The other data obtained from patients’ medical records were clinical side effects, including central nervous system status (CNS as yes or no), mediastinal tumor (yes/no), splenomegaly (yes/no), lymphadenopathies (yes/no), hepatomegaly (yes/no), rheumatoid arthritis signs (RA as yes or no), and other information. The outcome of interest in this study was time to relapse. Relapse was the event of interest, and death was the competing event because it precludes relapse. The patients who were lost to follow-up or did not experience any of these events were considered censored.

###  Cause-specific hazard model

 There are many clinical studies where the subjects may experience multiple types of events during the follow-up period, which are called competing risks.^26^ The cause-specific hazard model is one of the most widely used approaches to competing risk data. The cause-specific method is the typical approach that considers each type of event separately, whereas other types of competing events are considered censors.^8^ The cause-specific hazard model is especially useful and relevant when the etiology of the disease is of interest, as the event rate among the subjects who are at risk of developing the event of interest is quantified by this approach.^27^

 To investigate the effect of predictor variables on hazard function in the cause-specific hazard regression model, the Cox regression takes the following form^28^:


(1) $$h_{j}\left(t,X\right)=h_{0j}\left(t\right)\exp\left(\sum_{i=1}^{p}\beta_{ij}X_{i}\right)$$

In the above regression model, several types of events (j; j = 1, …, c) can occur, and *β*_ij_ are regression coefficients that show the effect of covariates on the j-th events, and p is the number of predictor variables in this model.

###  Mixture cure models

 Classical survival methods assume that every subject will eventually experience the event of interest, given sufficient follow-up time. However, there are some cases, especially with advances in modern medicine, where a proportion of subjects are “cured” and therefore will never experience the event of interest and will have long-term survival. Cure models should be used in these situations.^9^

 To estimate the parameters of the cure model, the follow-up time should be sufficiently long, and there should be a significant cure rate.^4^ In addition, one way to determine that a dataset may have a subset with longer survival is to examine the survival curve. If the survival curve at the end of the study is flat and parallel to the time axis, the cure model is a useful method for data analysis.^29^ One of the advantages of these models is that they take into account factors that affect both the survival function and the cure rate. Cure models include two general classes, namely, mixture and non-mixture models.^9^ The survival function for all subjects studied in this model is as follows:


(2) $$S_{POP}\left(t,X\right)=\left(1-\pi \right)+\pi S\left(t\left|Y=1,X\right.\right)$$

Let *t* denote the failure time for the event of interest, and *Y *is a binary latent variable, indicating whether a subject is susceptible (*Y* = 1) or immune (*Y* = 0), where (*Y* = 1) if the subject experiences the event of interest and (*Y* = 0) if the subject is cured. (1 – π) is the fraction of subjects who are cured, and *π* represents the incidence of being susceptible. In addition, *S (t |Y = 1*) is the survival function conditional on being susceptible, and the incidence is modeled using a logistic regression model:


(3) $$\pi \left(X\right)=p_{r}\left(Y=1\left|,X\right.\right)=\frac{\exp\left(X^{\prime }\beta \right)}{1+\exp\left(X^{\prime }\beta \right)}$$

Where *X* is a vector of covariates (including the intercept), and *β* denotes the vector of unknown coefficients.^10^

 The probability of cure for each person was calculated using Eq. (4), incorporating the coefficients derived from the fitted model and inputting each person’s characteristics into Eq. (4).^30^


(4) $$1-\hat{\pi }\left(X\right)=\frac{\exp\left(X^{\prime }\hat{\theta }\right)}{1+\exp\left(X^{\prime }\hat{\theta }\right)}$$

###  Penalized regression models 

 Penalized models are regression shrinkage and selection approaches that impose different penalties on the regression coefficients.^19,20^ Further, these methods have the oracle property; a model that includes only those factors with nonzero coefficients.^18^ In these methods, removing the predictor variables that are unrelated to the response variable increases the interpretability of the model and reduces the overfitting of the data.^31,32^

 The least absolute shrinkage and selection operator (LASSO) penalty was introduced by Tibshirani.^32^ It is defined as follows:


(5) $${\hat{\beta }}_{LASSO}=\arg\;\min_{\beta }(l\left(\beta \right)+\sum_{j=1}^{p}\lambda \left(\left|\beta_{j}\right|\right),$$

Where *l*(*β*) is the log-likelihood function, and *λ*(|*β*_j_|) represents the LASSO penalty function that depends on the tuning parameter. Furthermore, λ ≥ 0 denotes the tuning parameter that plays an important role in the selection of variables, and *β* = (*β*_1_*,…,β*_p_)^T^ is the vector of regression coefficients.

 The smoothly clipped absolute deviation (SCAD) penalty has been proposed by Fan and Li^31^ as follows:


(6) $${\hat{\beta }}_{SCAD}=\arg\;\min_{\beta }(l\left(\beta \right)+\sum_{j=1}^{p}p_{\lambda }\left(\left|\beta_{j}\right|\right)$$

(7) $${p^{\prime }}_{\lambda }\left(\left|\beta_{j}\right|\right)=\lambda \left\{I(\left|\beta_{j}\right|<\lambda )+\frac{\left(\alpha \lambda -\left|\beta_{j}\right|\right)}{\left(\alpha -1\right)\lambda }I\left(\left|\beta_{j}\right|\geq \lambda \right)\right\}$$

Where *l*(*β*) is the log-likelihood function, and *p*_λ_(|*β*_j_|) indicates the SCAD penalty function. Moreover, *λ* > 0 and *α* > 0 are tuning parameters, and ${p^{\prime }}_{\lambda }(\left|\beta_{j}\right|$ is the first derivative of the SCAD penalty function. Additionally, *β* = (*β*_1_*,…,β*_p_)^T^ is the vector of regression coefficients.

 For the applied method (i.e., the penalized mixture cure cause-specific competing risk model), the penalty terms were added to the likelihood function. Here, the LASSO and SCAD penalties were taken into consideration. The role of these penalties is to shrink the small coefficients toward zero.^10^ The estimates of the parameter vector can be obtained via an expectation-maximization algorithm. In this study, the tuning parameter *a* was considered 3.7,^10^ and the ones that minimize the Akaike information criterion (AIC) or the Bayesian information criterion (BIC) were selected for a set of possible values for *λ*.

###  Software

 In this study, all analyses of penalized mixture cure models in the presence of the competing risk model were performed using R software (version 4.3.1, URL: http://www.R-project.org) and the *penPHcure* package. The chi-square test, Fisher’s exact test, and Kruskal-Wallis test were used for univariate analysis.

## Results

 A total of 178 patients with ALL and AML participated in the study. The mean (standard deviation) age of the patients was 5.91 (3.86) years. The median (interquartile range) follow-up time was 67.33 (18.63, 99.57) months. The majority of patients (62%) did not experience any event (death or relapse) during the study period and survived until the final follow-up. However, 47 (25.1%) patients died before the relapse, and 24 (12.8%) patients experienced relapse. Overall survival rates at 1, 3, and 5 years were 93%, 89%, and 88%, and relapse-free survival rates were 79%, 75%, and 75%, respectively.

###  Demographic, clinical, and laboratory results

Table 1 provides the demographic, clinical, and laboratory characteristics of the patients. Most patients were male (55.1%) and aged 1‒9 years (79.1%), and the type of leukemia was ALL (86.1%).

 Based on the results, 158 (84.5%) patients had a PLT count lower than 150 000 cells/mL. In addition, 55 (29.4%) patients had a WBC count lower than 4000 cells/mL, and 93 (49.7%) patients had a WBC count higher than 11 000 cells/mL. Further, 178 (95.2%) patients had non-normal hemoglobin.

 The other characteristics of the patients are listed in Table 1. The type of leukemia (*P* = 0.009) and rheumatoid arthritis signs (*P* = 0.01) had a statistically significant difference between the groups. Furthermore, 16 (66.7%) of the patients who experienced a relapse and 40 (85.1%) of the patients who died had ALL.

**Figure 1:**
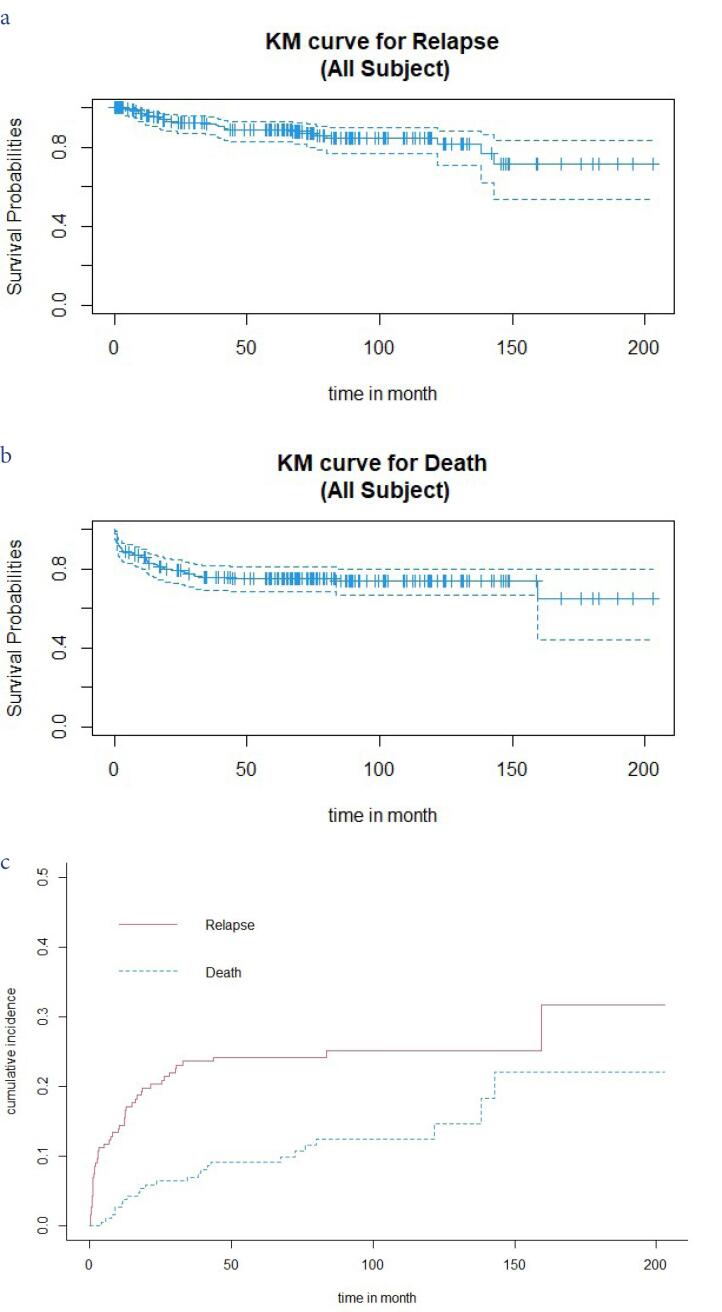
Kaplan–Meier survival curves for (a) relapse and (b) death, and (c) cumulative incidence for relapse and death for leukemia data. *Note*. KM: Kaplan–Meier

Figure 1 illustrates the Kaplan–Meier survival curve for relapse as the event of interest and death as a competing risk. As shown in Figure 1a, the survival curve at 150 months is flat, and the probability of survival is greater than 0.5. Moreover, the Kaplan–Meier plot depicts a clear plateau for relapse, which justifies the use of cure models. Figure 1b also displays the Kaplan–Meier survival curve for death. Additionally, the results of Maller and Zhou’s test revealed that there are patients with long-term survival in the present study. Figure 1c illustrates the cumulative incidence function for relapse and death. As shown, the cumulative incidence probability for relapse is greater than the competing event of death. Tables 2 and 3 summarize the estimated coefficients in the full model, including all 12 clinical and laboratory factors and the final models selected by the SCAD and LASSO penalty methods.

**Table 1:** Clinical and laboratory characteristics of patients and the comparison between right censored, relapsed, and death patients

**Continuous variables**	**Studied patients** **n=178 (100%)**		**Right censored** **n=116(62%)**		**Relapse** **n=24(12.8%)**		**Death** **n=47 (25.1%)**		***P*** ** value**
	**Mean**	**SD**	**Mean**	**SD**	**Mean**	**SD**	**Mean**	**SD**	
Age at diagnosis (y)	5.91	3.86	5.77	3.70	5.01	2.93	6.71	4.53	0.490
Categorical variables	**Number**	**%**	**Number**	**%**	**Number**	**%**	**Number**	**%**	***P*** **-value**
Gender									0.790
Female	84	44.9	51	44.0	10	41.7	23	48.9	
Male	103	55.1	65	56.0	14	58.3	24	51.1	
Type of leukemia									0.009
AML	26	13.9	11	9.5	8	33.3	7	14.9	
ALL	161	86.1	105	90.5	16	66.7	40	85.1	
WBC count									0.350
< 4000	55	29.4	39	33.6	6	25.0	10	21.3	
4000-11 000	39	20.9	25	21.6	6	25.0	8	17.0	
> 11 000	93	49.7	52	44.8	12	50.0	29	61.7	
Hb count									0.540
Normal	9	5.0	5	4.3	2	8.3	2	4.3	
Ab normal	178	95.0	111	95.7	22	91.7	45	95.7	
PLT count									0.250
< 150 000	158	84.5	97	83.6	22	91.7	39	83.0	
150 000-400 000	21	11.2	21	12.9	0	0.0	6	12.8	
> 400 000	8	4.3	4	3.4	2	8.3	2	4.3	
CNS status on diagnosis									0.810
Yes	5	2.7	3	2.6	1	4.2	1	2.1	
No	182	97.3	113	97.4	23	95.8	46	7.9	
Mediastinal tumor									0.080
Yes	14	7.5	5	4.3	3	12.5	6	12.8	
No	173	92.5	111	95.7	21	87.5	41	87.2	
Splenomegaly									0.190
Yes	101	54.0	57	49.1	16	66.7	28	59.6	
No	86	46.0	59	50.9	8	33.3	19	40.4	
Lenfadenopati									0.590
Yes	96	51.3	62	53.4	10	41.7	24	51.1	
No	91	48.7	54	46.6	14	58.3	23	48.9	
Hepatomegaly									0.910
Yes	84	44.9	52	44.8	10	41.7	22	46.8	
No	103	55.1	64	55.2	14	58.3	25	53.2	
Rheumatoid arthritis signs									0.010
Yes	102	54.5	70	60.3	15	62.5	17	36.2	
No	85	45.5	46	39.7	9	37.5	111	63.8	

*Note*. SD: Standard deviation; AML: Acute myelogenous leukemia; ALL: Acute lymphocytic leukemia; WBC: White blood cells, Hb: Hemoglobin; PLT: Platelets; CNS: Central nervous system

###  Results of the full PH-Cox mixture cure competing risk model

Table 2 presents the results of the full PH-Cox mixture cure model under competing risks (with all variables present) with the enter method using a cause-specific approach. Based on the findings, none of the factors were statistically significant for the subjects with long-term (cure coefficient estimates) and short-term (survival coefficient estimates) survival, and the 95% confidence interval for the factors included zero; therefore, no factors were detected in this model without consideration of the penalty function when the event of interest is relapse. The clinical factors PLT and lenfadenopati in the latency component event were statistically significant for death, and the 95% confidence interval did not include zero. The risk of death was higher in patients with PLT counts greater than 400 000 cells/mL than in patients with PLT counts between 150 000 and 400 000 cells/mL (β = -2.21, hazard ratio (HR) = 0.1). In addition, the risk of death was 2.58 times higher for patients with lymphadenopathies than for patients without lymphadenopathies $\left(\beta =0.95,\;HR=2.58\right).$ Confidence intervals were calculated using the bootstrap method with 1000 replications.

**Table 2:** Identification of important prognostic factors affecting short-term and long-term survival of leukemia patients using the full PH-cox mixture cure competing risk model

**Prognostic factors**	**Event=Relapse and competing risk=Death**		**Event=Death and competing risk=Relapse**	
	**Cure (incidence)** **coefficient estimates** **(95% CI)**	**Survival (latency)** **coefficient estimates** **(95% CI)**	**Cure (incidence)** **coefficient estimates** **(95% CI)**	**Survival (latency)** **coefficient estimates** **(95% CI)**
Age at diagnosis (y)	-0.09 (-0.69, 0.31)	-0.20 (-1.85, 2.78)	0.11 (-0.63, 1.95)	-0.03 (-0.33, 0.12)
Female/Male (ref.)	0.58 (-0.95, 3.901)	-0.60 (-13.23, 28.63)	-0.05 (-1.91, 31.15)	0.23 (-1.39, 1.65)
ALL/AML (ref.)	-1.61 (-3.21, 9.04)	-2.54 (-17.18, 23.99)	-1.35 (-6.23, 66.07)	1.55 (-0.80, 5.09)
WBC count < 4000 / > 11 000 (ref.)	0.80 (-2.14, 4.57)	-4.28 (-13.07, 47.06)	-0.64 (-6.27, 5.00)	-0.96 (-2.11, 1.36)
4000-11 000 / > 11 000 (ref.)	1.07 (-42.74, 3.18)	-1.99 (-8.84, 21.70)	0.009 (-75.86, 1.56)	-0.42 (-3.20, 1.62)
Hemoglobin, normal/Ab normal (ref.)	2.20 (-43.43, 38.07)	-1.81 (-33.38, 24.13)	35.60 (-24.60, 89.17)	-2.20 (-7.80, 30.45)
Platelets, < 150 000 / > 400 000 (ref.)	0.33 (-30.54, 37.80)	-0.38 (-36.36, 24.69)	1.12 (-81.30, 36.66)	-1.52 (-35.71, 1.07)
150 000-400 000 / > 400 000 (ref.)	-32.51 (-50.30, 37.49)	-28.20 (-50.14, 38.09)	2.54 (-142.46, 44.33)	-2.21 (-37.21, -0.12)
CNS, Yes/No (ref.)	1.84 (-35.389, 39.54)	-4.19 (-11.32, 41.62)	-0.86 (-8.57, 71.25)	1.80 (-1.80, 35.90)
Mediastinal tumor, Yes/No (ref.)	0.65 (-46.77, 28.73)	4.93 (-45.07, 34.65)	1.94 (-36.94, 12.86)	-0.41 (-3.33, 0.63)
Splenomegaly, Yes/No (ref.)	2.23 (-60.34, 4.63)	0.29 (-40.68, 9.15)	0.64 (-39.80, 3.20)	-1.04(-3.40, 0.50)
lymphadenopathies, Yes/No (ref.)	0.52 (-3.56, 3.09)	1.85 (-13.00, 30.81)	0.95 (-26.41, 3.22)	0.95 (0.18, 3.21)
Hepatomegaly, Yes/No (ref.)	-2.07 (-4.60, 48.67)	1.82 (-18.18, 29.89)	0.27 (-1.13, 29.67)	-0.37 (-2.51, 0.91)
Rheumatoid arthritis signs, Yes/ No (ref.)	1.06 (-5.98, 4.44)	0.34 (-17.32, 22.27)	1.01 (-16.96, 3.92)	1.15 (-2.33, 1.48)

*Note*. CI: Confidence interval; AML: Acute myelogenous leukemia; ALL: Acute lymphocytic leukemia; WBC: White blood cells; CNS: Central nervous system.

###  Results of the penalized PH-Cox mixture cure competing risk model

Table 3 (sections a and b) reports the results of variable selection in the PH-Cox mixture cure competing risk model with a cause-specific approach using SCAD and LASSO penalty methods. Variable selection was performed using BIC and AIC criteria.

**Table 3:** Identification of important prognostic factors affecting short-term and long-term survival of leukemia patients using the penalized PH-cox mixture cure competing risk model

	**SCAD penalty**	**LASSO penalty**
**A: Event=Relapse and competing risk=Death**	**Coefficient estimates**	**Coefficient estimates**
Cure (incidence)		
Intercept	-0.34	-0.75
Platelets 150 000-400 000	-1.14	0.00
Platelets > 400 000	Ref.	Ref.
Relapse-specific hazard (latency)		
Acute lymphocytic leukemia	-2.50	-0.81
Acute myelogenous leukemia	Ref.	Ref.
Mediastinal tumor: Yes	2.79	0.00
Mediastinal tumor: No	Ref.	Ref.
Splenomegaly: Yes	1.08	0.00
Splenomegaly: No	Ref.	Ref.
**B: Event=Death and competing risk=Relapse**		
Cure (incidence)		
Intercept	-0.37	-0.37
death-specific hazard (latency) component		
WBC < 4000	-1.37	-0.28
WBC > 11 000	Ref.	Ref.
Rheumatoid arthritis signs: Yes	1.75	0.77
Rheumatoid arthritis signs: No	Ref.	Ref.

*Note*. SCAD: Smoothly clipped absolute deviation; LASSO: Least absolute shrinkage and selection operator; WBC: White blood cell.

 The interpretation of the results has been reported in cure and latency components. The coefficients for the cure component are denoted by θ and estimated using a logistic regression model, whereas the coefficients for the latent component are represented by β and estimated using the Cox model.

 Based on the SCAD-penalized method (Table 3, section a), the clinical factor PLT was selected in the cure (incidence) part. The negative sign of the estimated coefficient implies that subjects with PLT between 150 000 and 400 000 versus more than 400 000 were less susceptible to relapse (*θ* = -1.14, OR = 0.31). The type of leukemia, mediastinal tumor, and splenomegaly factors were selected in the relapse-specific hazard (latency) component. The negative sign of the estimated coefficient for the type of leukemia implies that subjects with ALL had a lower risk of relapse compared to those with AML (*β* = -2.5, HR = 0.08). Conversely, the positive coefficients for mediastinal tumor (*β* = 2.79, HR = 16.28) and splenomegaly (*β* = 1.08, HR = 2.94) indicated an increased risk of relapse among susceptible subjects. In essence, the risk of relapse in AML patients was 12.5 (1/exp (*β*)) times higher than in ALL patients. Moreover, the risk of relapse for patients with mediastinal tumors and splenomegaly was 16.28 and 2.94 times higher than for patients without these conditions, respectively.

 Using the SCAD-penalized method, the WBC and rheumatoid arthritis signs were identified in the death-specific hazard (latency) component (Table 3, section b). The risk of death in patients with WBCs was over 11 000 cells/mL, 4 times higher than in patients with WBCs less than 4000 cells/mL (β = -1.37, HR = 0.25). Additionally, the risk of death for patients with rheumatoid arthritis signs was 5.75 times higher than for patients without these signs (β = 1.75, HR = 5.75).

Table 4 provides the BIC and AIC criteria of the penalized PH-Cox mixture cure competing risk model and the full model. Based on the data, the BIC and AIC criteria of the PH-Cox mixture cure competing risk with the SCAD penalty were lower than the LASSO penalty and the full model.

**Table 4:** Comparison of the models’ performance based on the AIC and BIC criteria

**Method**	**Event=Relapse and competing risk=Death**		**Event=Death and competing risk=Relapse**	
	**BIC**	**AIC**	**BIC**	**AIC**
SCAD	314.59	298.23	477.75	468.06
LASSO	322.61	308.82	486.50	472.51
Full model	332.62	319.08	517.24	480.72

*Note*. BIC: Bayesian information criterion; AIC: Akaike information criterion; SCAD: Smoothly clipped absolute deviation; LASSO: Least absolute shrinkage and selection operator.

## Discussion

 Traditional time-to-event survival models often consist of one event. However, in healthcare studies, a patient may experience multiple clinical events instead of a single event.^8,28^ The occurrence of competing events may preclude the event of interest; therefore, typical survival data analysis cannot be used as it may produce misleading results. The cause-specific approach to analyzing competing risk data involves performing a separate survival analysis for each event. Other (competing) events are treated as censoring. This method requires the assumption that competing risks are independent. Currently, no method exists that can directly assess independence or guarantee correct estimates when it is violated. Therefore, survival analysis assumes that the independence assumption for competing risks exists even if it does not. The only indirect method is to perform a “sensitivity analysis”.^33^

 In some clinical studies, a group of patients may respond to treatment and become immune to the disease. They commonly have long-term survival and are cured. The rest of the patients will not be cured and will experience an event of interest if there is sufficient follow-up.^18^ In these studies, several factors may be measured over time, and it is important to identify factors that affect the long-term and short-term survival of the subjects. Researchers are interested in selecting important factors for the failure time distribution of uncured patients and for the cured part.^10,18^ Sometimes, the researchers have encountered types of data that include both cure rate and competing risk features simultaneously, and it is important to identify prognostic factors for the disease. Few studies have been conducted in the context of variable selection for cured competing risk data.

 In this study, a penalty-based variable selection method was applied for the semi-parametric mixture cure model in the presence of competing risks using LASSO and SCAD penalties. The findings revealed that PLT was chosen in the cured part, while the leukemia type, mediastinal tumor, and splenomegaly were selected in the relapse-specific hazard part. Furthermore, the results indicated that the WBC and rheumatoid arthritis signs were selected in the death-specific hazard part.

 Based on the results (Table 2), the estimate for hemoglobin was calculated as large, and as a result, the estimate of the hazard ratio was also large. This issue occurs due to sparse data. In this study,the number of individuals with normal hemoglobin levels in patients who relapsed and died was small and sparse data existed. The sparse data led to inflated standard deviations and invalid coefficient estimates in regression models. One solution to this problem is to use a Bayesian approach and regularization methods.^34,35^

 Some studies have been performed to identify significant prognostic factors for relapse and death in leukemia patients, where relapse and death were considered the event of interest and a competing risk, respectively. The main limitation of these studies is that none of them considered the characteristic of the patient’s cure rate. The results of these studies demonstrated that some laboratory and demographic factors, such as age, gender, hemoglobin level, PLT, WBC, and central nervous system, have been identified as prognostic factors for relapse and death.^36-38^

 The results of a study by Bhojwani et al revealed that age at diagnosis, WBC, PLT, and hemoglobin levels were all prognostic factors for both death and relapse in children with leukemia. The presence of central nervous system disease at diagnosis and early response to treatment also had a significant impact on the risk of relapse. However, gender and race were not found to be significant factors for either death or relapse.^39^ Based on the findings of another study by Hunger et al, age, gender, and WBC were reported as significant factors of death. In our study, WBC and rheumatoid arthritis signs were found to be significant factors for death.^40^ The levels of WBC, PLT, and hemoglobin are important factors in both relapse and death in children with leukemia. These levels indicate the extent of bone marrow infiltration by leukemic cells and the degree of bone marrow suppression caused by the disease and its treatment. Higher WBC and lower PLT and hemoglobin levels are associated with more advanced and aggressive disease, which increases the risk of relapse and death. Therefore, clinicians should monitor these hematologic parameters to identify patients at higher risk of relapse and death and to guide treatment decisions for optimal patient outcomes.^40-42^

 It is important to note that this study had some limitations. First, the data used in the study were collected retrospectively from patients’ medical records, implying that some factors were not recorded. Therefore, the study could not examine the effect of other influential variables. Accordingly, it is suggested that prospective cohort studies be conducted using the applied model to identify risk factors for relapse and death in leukemia patients. Second, the sample size utilized in the study was small. Nonetheless, we used an advanced statistical method to identify factors associated with relapse and death using a cause-specific approach. According to Tibshirani, the penalized methods can be applied in many situations, even when the number of factors is smaller than the sample size.

**Highlights**

- This study assessed prognostic factors of patient survival and relapse using a penalized semi-parametric mixture cure competing risk model.
- Platelet levels, leukemia type, mediastinal tumor, and splenomegaly were significant in predicting relapse, while white blood cell count and rheumatoid arthritis signs were linked to death.
- These findings underscore the importance of laboratory and clinical factors in managing childhood leukemia outcomes.

## Conclusion

 In this study, a new approach was employed for variable selection in a semi-parametric competing risk cure model. According to the BIC and AIC criteria and the better performance of the SCAD penalized method, our findings indicated that factors such as PLT, type of leukemia, mediastinal tumor, and splenomegaly have an impact on the risk of leukemia relapse. It is suggested that oncologists pay attention to these prognostic factors during the screening period of these patients.

## Acknowledgements

 This study was part of a Ph.D. thesis in Biostatistics. We would like to appreciate the Vice-Chancellor of Education of Hamadan University of Medical Science for technical support for the approval and support of this study.

## Authors’ Contribution

**Conceptualization:** Leili Tapak, Zahra Mehrbakhsh.

**Data curation:** Nasser Behnampour.

**Formal analysis:** Zahra Mehrbakhsh, Leili Tapak.

**Funding acquisition:** Ghodratollah Roshanaei.

**Investigation:** Zahra Mehrbakhsh, Leili Tapak, Ghodratollah Roshanaei.

**Methodology:** Zahra Mehrbakhsh, Leili Tapak.

**Project administration:** Ghodratollah Roshanaei, Leili Tapak.

**Resources:** Ghodratollah Roshanaei.

**Software:** Zahra Mehrbakhsh, Leili Tapak.

**Supervision:** Ghodratollah Roshanaei, Leili Tapak.

**Validation:** Ghodratollah Roshanaei.

**Visualization:** Ghodratollah Roshanaei.

**Writing–original draft:** Zahra Mehrbakhsh, Leili Tapak, Ghodratollah Roshanaei, Nasser Behnampour.

**Writing–review & editing:** Zahra Mehrbakhsh, Leili Tapak, Ghodratollah Roshanaei.

## Competing Interests

 The authors declare no competing interests.

## Ethical Approval

 This study was approved by the Ethics Committee of Hamadan University of Medical Sciences (Approved Ethical Code: IR.UMSHA.REC. 1400.411). Informed written consent was obtained from the parent or legal guardian of each study participant.

## Funding

 This study is the result of a Ph.D. thesis by Zahra Mehrbakhsh, which was supported by the Vice Chancellor for Research and Technology of Hamadan University of Medical Sciences, Iran (Grant No. 140006094622).
